# Supplementary material for: Timing of Pathogen Adaptation to a Multicomponent Treatment
Source: PLoS One. 2013 Aug 21;8(8):e71926. doi: 10.1371/journal.pone.0071926 (PMC3749216; doi:10.1371/journal.pone.0071926)
Supplement: Supporting Information S1 — The distribution law of the emergence time (7). The model shows that for strategies Str5 and Str4, when the number of treatment components, , increases, the law of the emergence time approaches an exponential one. Conversely, when the number of treatment components is small, the law moves away from an exponential distribution and towards a normal one. (PDF) [file pone.0071926.s001.pdf]

Supporting Information S1: Emergence time law

R. Bourget*, L. Chaumont, N. Sapoukhina
∗ E-mail: bourget@math.univ-angers.fr

To compare the durability of the treatment strategies, it suffices to estimate the mean emergence time of a pathogen mutant deafiting the multicomponent treatment and of its variance. Nevertheless, the model and numerical simulations make it possible to obtain the distribution law of the emergence time S (7). The model shows that for strategies Str5 (Fig. S1A-C) and Str4 (not illustrated), when the number of treatment components, N, increases, the law of the emergence time approaches an exponential one. Conversely, when the number of treatment components is small, the law moves away from an exponential distribution and towards a normal one. In the case of strategies Str1 (Fig. S1D-F), Str2 and Str3 (not illustrated), the law of S is normal. These results remain valid for the other parameter couples $(r, D) = (0.3, 0.3)$ and $(12, 0.1)$.

Our results demonstrate that the law of the emergence time depends on the host population structure resulting from the treatment strategy deployed. If hosts carrying intermediate treatments are available for pathogens, the emergence time is well distributed around its mean. This can be explained by the fact that each mutation can be easily fixed within the hosts receiving intermediate treatments. In contrast, in strategy Str5, the absence of intermediate treatments makes it difficult for mutations to accumulate. So even if several mutations appear, they can easily disappear, and then the mutation process has to restart in order to produce a mutant able to infect hosts receiving a multicomponent treatment. This prolongs the emergence time, leading to the exponential law. This phenomenon intensifies with the increase of the number of treatment components. This finding can be related to those of [1]. Indeed, in case of strategies Str5 and Str4, the higher the number of treatment components, the closer the initial conditions are to those that result in the exponential distribution of S.

References

1. Bourget R (2013) Modélisation stochastique des processus d'adaptation d'une population de pathogène aux rsistances génétiques des hôtes. Ph.D. thesis, Université d'Angers.

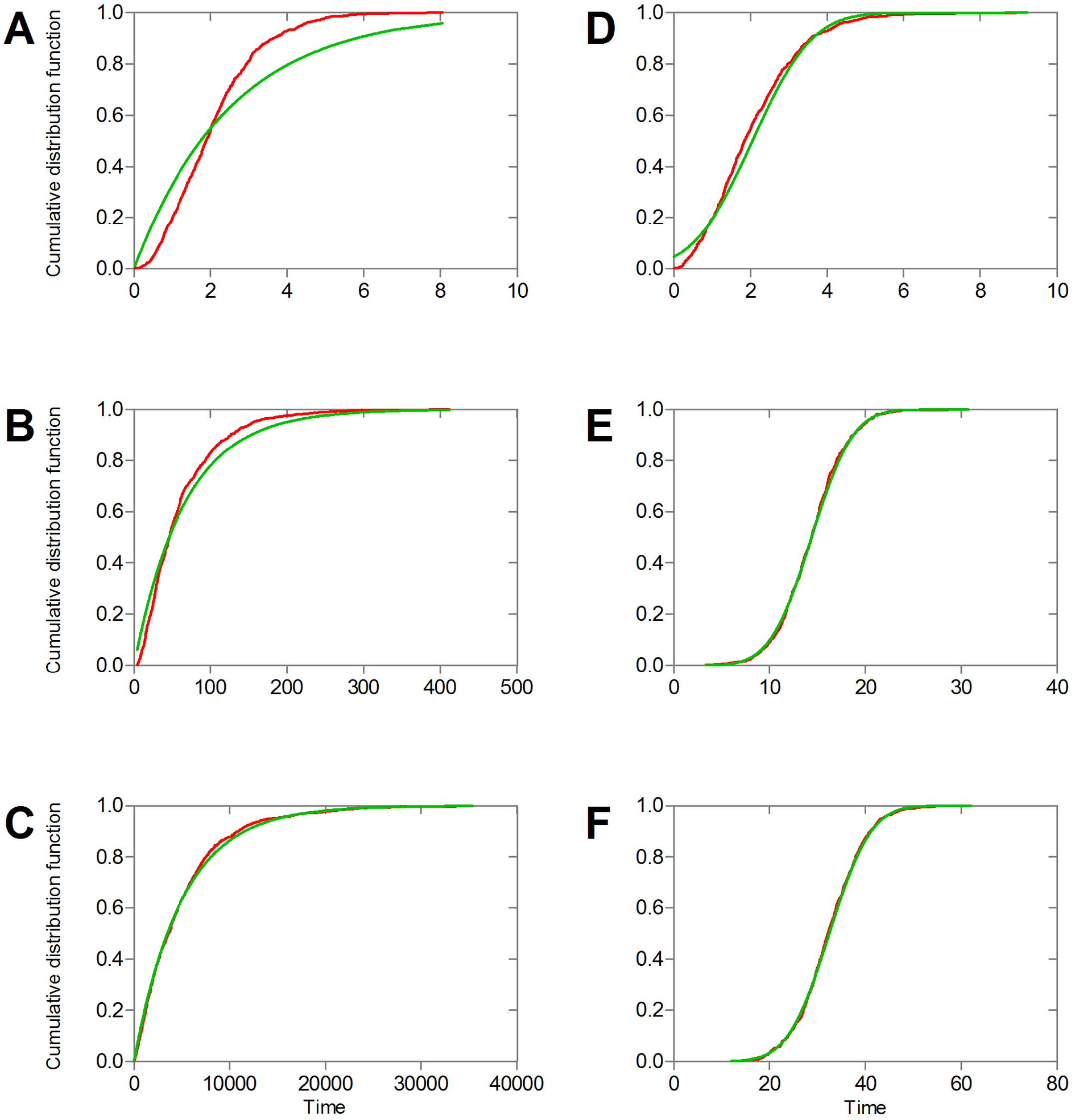

Figure S1. Emergence time law. *Cumulative distribution function of* S *(7) for two different strategies (Str5 for (A-C) and Str1 for (D-F)) in red, and cumulative distribution function of ordinary laws in green, where the parameters are set to obtain the smallest sum of squared residuals between the two functions: an exponential law with parameters* λ_T *for (A-C), and the normal law with mean* μ_S *and standard deviation* σ_S *for (D-F). The number of treatment components is as follows* $N =$ *(A,D) 1, (B,E) 2 and (C,F) 3. We find for the exponential laws* $\lambda_S =$ *(A)* 0.397*, (B)* 0.015 *and (C)* 0.0002*, and for normal laws* $(\mu_S, \sigma_S) =$ *(D) (2.05,1.22), (E) (14.38,3.39) and (F) (32.45,6.78). Other parameters are fixed to* $r = 1$*,* $D = 0.2$ *et* $K = 10000$.
