# Supplementary material for: Timing of Pathogen Adaptation to a Multicomponent Treatment
Source: PLoS One. 2013 Aug 21;8(8):e71926. doi: 10.1371/journal.pone.0071926 (PMC3749216; doi:10.1371/journal.pone.0071926)
Supplement: Supporting Information S2 — A variance-based global sensitivity analysis of model (1–6). We find that all parameters and almost all the interactions between them have a significant effect on the emergence time S. (PDF) [file pone.0071926.s002.pdf]

Supporting Information S2: Sensitivity Analysis

R. Bourget*, L. Chaumont, N. Sapoukhina
* E-mail: bourget@math.univ-angers.fr

To investigate the impact of interactions between parameters on the model behaviour and rank parameters by influence, we conducted a variance-based global sensitivity analysis of model (1-6). We performed an analysis of variance (ANOVA) of the emergence times S (7) obtained for a complete factorial design including 6 parameters (ν, K, C, D, r, ξ), and for the strategy Str5 with a number of component treatments $N = 1$. To build the complete factorial design we used two extreme values of the parameters' ranges: $\nu = 10^{-5}$ and 10^{-3}, $K = 1000$ and 10000, $C = 0$ and 0.8, $D = 0.1$ and 0.3, $r = 0.3$ and 12, and $\xi = 0.2$ and 0.8. Parameter ranges were defined from empirical studies (Table 1) or expert opinions. Thus, 2^6 parameter combinations were tested.

To compare the contribution of the different parameters and their interactions to the total variability, we used sensitivity indices that can be obtained by decomposing the response variability (SS_T) into factorial terms as follows [1, 2] :

$$SS_T = \sum_i SS_i + \sum_{i<j} SS_{ij} + \cdots + SS_{1\ldots6},$$

including main effects (SS_i) and interactions between up to 6 factors ($SS_{1\ldots6}$). The main effects sensitivity indices are the proportions of the response variability that are explained by parameter i, SS_i/SS_T. Interaction sensitivity indices are the proportions of the response variability that are explained by interactions between up to 6 parameters, $SS_{i\ldots j}/SS_T$. Total sensitivity indices are the proportions of response variability explained by the main effects and all interactions related to a particular parameter i,

$$\frac{SS_i + \sum_{j\neq i} SS_{ij} + \cdots + SS_{1\ldots6}}{SS_T}.$$

We find that all parameters and almost all the interactions between them, except $K : \nu : r : \xi$ (p-value = 0.068), $C : D : \nu : r : \xi$ (p-value = 0.079), $C : D : r : \xi$ (p-value = 0.083), $C : D : K : r : \xi$ (p-value = 0.098) and $C : D : K : \nu : r : \xi$ (p-value = 0.12), have a significant effect on the emergence time S. Figure S2 shows that the parameters that explain most of the variability are the mutation rate, ν, and the maximum pathogen population size, K. This result was expected, since these parameters have a direct impact on the probability that a mutant individual will appear. The remaining variability is explained, in descending order, by the mutation cost C, the migration rate D, and the reproduction rate r. Finally, the lowest contribution to the emergence time variability was the proportion of the host treated, ξ. Since this parameter has a nonlinear effect (see Fig. 1), the two-level factorial design might

be not sufficient to capture its effect.

Interactions between parameters with the highest main-effect, $K : \nu$, make the greatest contribution to the variability. They are followed by interactions including ν or K, e.g. $C : \nu$, $K : D$, $C : K : \nu$, etc. Interactions with the lowest contribution are those including ξ. Nevertheless, Figure S2 shows that, even if the main-effect of C explains more variability than the main-effect of D, interactions with D explain more variability than interactions including C.

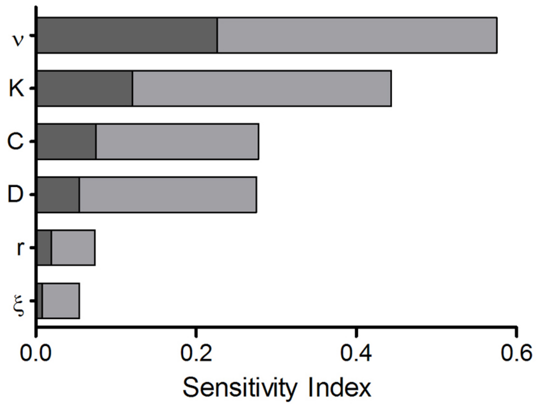

Figure S2. Main-effect (first part of the bars) and total (full bars) sensitivity indices. Indices are based on the complete factorial design (2^6) and its analysis of variance, for model (1-6).

References

1. Saltelli A, Campolongo F, Tarantola S, Ratto M (2004) Sensitivity Analysis in Practice: A Guide to Assessing Scientific Models. Wiley.

2. Monod H, Naud C, Makowski D (2006) In: Wallach D, Makowski D, Jones J, editors, Working with Dynamic Crop Models: Evaluation, Analysis, Parameterization, and Applications, Elsevier Science.
